# Subdural Pressure and Brain Condition During Propofol Vs Isoflurane - Nitrous Oxide Anaesthesia in Patients Undergoing Elective Supratentorial Tumour Surgery

**Published:** 2009-02

**Authors:** Sankari Santra, Bibhukalyani Das

**Affiliations:** 1 Assistant Professor, Department of Neuro Anaesthesiology, Neuro ICU and Pain Clinic, Bangur Institute of Neurosciences & Psychiatry, Kolkata, INDIA; 2 Professor, Department of Neuro Anaesthesiology, Neuro ICU and Pain Clinic, Bangur Institute of Neurosciences & Psychiatry, Kolkata, INDIA

**Keywords:** Subdural pressure, Cerebral perfusion pressure, Propofol, Isoflurane- nitrous oxide, Hyperventilation, Supratentorial tumour

## Abstract

**Summary:**

Total intravenous anaesthesia has received much importance than inhalational anaesthesia in neuroanaesthetic practice. In an effort to determine whether any important clinical differences occur, studies concerning intracranial pressure (ICP), degree of dural tension and degree of brain swelling during intravenous and inhalational based anaesthesia are warranted like the present one.

A total of 68 patients were assigned randomly to one of two groups. In Group-I(n=34), anaesthesia was induced with propofol (1-3mg.kg^−1^) and maintained with propofol (6-10mg.kg^−1^.hr^−1^) and fentanyl (2-3mcg.kg^−1^.hr^−1^). In Group-II (n=34), anaesthesia was induced with propofol (1-3mg.kg^−1^) but maintained with isoflurane, nitrous oxide and fentanyl (2-3mcg.kg^−1^.hr^−1^). Moderate hypocapnia was applied to maintain arterial carbon dioxide around 30mmHg. Mean arterial blood pressure was stabilized with phenylephrine whenever necessary. Subdural intracranial pressure, mean arterial pressure, cerebral perfusion pressure were monitored before and after 10min period of hyperventilation. Furthermore, the tension of dura before and after of hyperventilation and the degree of brain swelling after opening of dura were also estimated by the neurosurgeon.

No differences were found between the groups with regards to demographics, neuroradiologic diagnosis, position of head and time of ICP measurement. Before hyperventilation, both ICP and dural tension were significantly lower in Group I compared with Group-II (*P*<0.05). But after hyperventilation there was no significant difference of ICP and dural tension in between groups. The degree of brain swelling after opening of dura was similar in both groups. There was a positive correlation between measured ICP and brain swelling score.

## Introduction

Major goals of neurosurgical anaesthesia are maintenance of haemodynamic stability, sufficient cerebral perfusion pressure, relaxed brain to facilitate neurosurgical resection and avoidance of agents or procedures that increase intracranial pressure (ICP).

There has been long standing controversy regarding use of inhalational or intravenous anaesthetic agent for intracranial procedure. Total intravenous anaesthesia has always received much importance to avoid cerebral vasodilating effect of nitrous oxide (N_2_O) and volatile agents. But so far no study comparing intravenous with volatile based anaesthesia has been able to demonstrate major outcome difference^1^,^2^. However most of these studies examined a heterogenous patient population and ICP measured by different techniques.

Several experimental and clinical studies of cerebral haemodynamics including cerebral blood flow (CBF), cerebral metabolism for oxygen (CMRO_2_), ICP have been conducted during isoflurane^3^,^4^, propofol^5^–^7^ anaesthesia. Only few comparative studies of ICP are available. In one prospective trial, where three anaesthetic techniques (isoflurane-N_2_O, N_2_O-fentanyl, propofol-fentanyl) were used for elective supratentorial craniotomy, epidural ICP was measured through burr hole. Though ICP did not differ significantly, more patients with isoflurane- N_2_O group had ICP greater than 24mmHg as compared to other two groups^1^. Other studies monitoring lumbar cerebrospinal fluid (CSF) pressure demonstrated conflicting results, either no difference of ICP during propofol versus thiopentoneisoflurane anaesthesia^2^ or significantly lower ICP during propofol compared to isoflurane^8^.

During craniotomy one of the most critical point is opening of dura where a high ICP may cause some degree of brain swelling^9^. Subdural ICP measurement after removal of bone flap is a regional estimate of ICP which is influenced by presence of space occupying lesion (SOL)^10^ and gravity^11^. In one study, the level of subdural ICP and intraventricular pressure correlated well with the dural tension and the degree of brain swelling after opening of dura, estimated by neurosurgeons, blinded to the level of ICP^12^. In a recent trial, subdural ICP and incidence of brain swelling after opening of dura were significantly lower during propofol anaesthesia when compared to isoflurane and sevoflurane.^13^

The primary goal of present study was to detect any difference in subdural ICP occured during propofol versus isoflurane-nitrous oxide anaesthesia along with the effect of hyperventilation in patients undergoing elective craniotomy for supratentorial tumour. Degree of dural tension and brain swelling after opening of the dura were also studied as secondary objectives.

## Methods

The study was undertaken at Bangur Institute of Neuroscience and Psychiatry after approval of the protocolby local ethical committee. It was a prospective randomized trial. Verbal and written consent was taken from all patients.

Sixty eight patients of age 18 to 65 years, of either sex, American Society of Anesthesiologists physical status I and II, Glasgow Coma Scale (GCS) of 15 undergoing elective craniotomy for supratentorial tumour resection were randomly allocated in one of the two groups. Group I- Propofol (n= 34), Group II-Isoflurane-nitrous oxide (n=34).

Patients diagnosed as having supratentorial tumour with midline shift of less than 10mm (revealed by cerebral computed tomography or magnetic resonance imaging) were included in this study. Medically controlled hypertension or diabetes mellitus were also included.

Patients were excluded if they suffered from ischaemic heart disease, congestive heart failure, renal or hepatic dysfuntion, or severe chronic respiratory disease.

## Anaesthesia and monitoring

The patients were premedicated with 150mg oral ranitidine one hour prior to anaesthesia. Preoperative corticosteroid, anticonvulsant, antihypertensive were administered as usual. Monitoring before induction consisted of automated noninvasive blood pressure, continuous electrocardiogram and pulse oximetry. After induction of anaesthesia, a radial artery catheter was inserted with zero pressure adjustment at mid-axillary line for continuous blood pressure monitoring and blood sampling. Urine output and rectal temperature were continuously monitored throughout the procedure. Inspired and end-tidaloxygen, carbon dioxide, nitrous oxide and isoflurane were measured. Lungs were mechanically ventilated to maintain an arterial blood carbon-dioxide tension between 30-40 mmHg and inspiratory peak pressure less than 20cm of H_2_O. Train offour was used to monitor muscular relaxation which was achieved by acontinuous infusion of atracurium. The anaesthetic procedures were as follows.

## Group–I: Propofol

Anaesthesia was induced with 1-3mg.kg^−1^ propofol over 1min and 2-4mcg.kg^−1^ fentanyl. Lidocaine 1mg.kg^−1^ was administered over 1 minute followed by muscle relaxation with 0.5mg.kg^−1^ atracurium. After 3 minutes of mask ventilation with 100% oxygen, trachea was intubated. Lungs were mechanically ventilated with oxygen in air (fraction of oxygen 0.5). Anaesthesia was maintained with infusion of propofol (6-10mg.kg^−1^.hr^−1^) and fentanyl (2-3mcg.kg.^−1^hr^−1^). Rate of propofol infusion was adjusted to maintain adequate depth of anaesthesia. Just before skin incision of scalp, fentanyl 1mcg.kg^−1^ was supplemented. Infusion rate of propofol and fentanyl were kept unchanged during ICP measurement and during estimation of dural tension.

## Group–II:Isoflurane-nitrous oxide

Anaesthesia was induced with 1-3 mg.kg^−1^ propofol over 1min and 2-4mcg.kg^−1^ fentanyl. Lidocaine 1mg.kg^−1^ was administered over 1minute followed by muscle relaxation with 0.5mg.kg^−1^ atracurium. After 3 minutes of mask ventilation with 100% oxygen, trachea was intubated. Lungs were mechanically ventilated with 50% nitrous oxide in oxygen (fraction of oxygen 0.5). Isoflurane was added to the inspired gas mixture and concentration was increased in 0.2% increments for maintaining adequate depth of anaesthesia. Fentanyl infusion was administered (2-3mcg.kg^−1^.hr^−1^) as in Group-I. Just before skin incision of scalp fentanyl 1mcg.kg^−1^ was supplemented. Infusion rate of fentanyl and percentage of isoflurane were kept unchanged during ICP measurement and during estimation of dural tension.

Intravenous phenylephrine was administered if systolic blood pressure decreased greater than 20mmHg of baseline inspite of normal saline infusion. Mannitol 0.75gm.kg^−1^ was administered to all patients while making first burr hole.

### Subdural Intracranial Pressure measurement, Estimation of Dural tension and Brain swelling:

After removal of bone flap, a 22G/0.8mm venflon cannula was placed under dura and connected to a pressure transducer system via a polyethylene catheter. Zero level of ICP was adjusted with the transducer kept at the level of orbitomeatal line. After 1minute of stabilization mean value of subdural pressure was used as an estimate of ICP. After initial measurement of ICP, pulmonary ventilation was increased by 30% (increasing rate and tidal volume) for 10min. Subdural ICP was again measured at 11^th^ min after first measurement. Cerebral perfusion pressure (CPP) was calculated as the difference between mean arterial pressure (MAP) and ICP.

Estimation of dural tension was made in a scale of four, using tactile evaluation by neurosurgeon. Neurosurgeons were blinded to anaesthetic technique. The tension was categorized as follows: (1) very slack (2) normal (3) increased tension (4) pronounced increased tension. The degree of brain swelling was evaluated by the neurosurgeon after opening of the dura. Degree of swelling was estimated and categorized as follows (1) no swelling, excellent operating condition (2) minimum swelling (3) moderate swelling and (4) pronounced swelling of the brain.

## Statistical analysis

Based on a previous study of ICP, given a minimal detectable significant difference of 3.5mmHg, expected SD 5.0mmHg, power of 0.80, and a statistical significance level of *P*<0.05, the total sample size (number of patients) was calculated to be 68. Data within groups were tested for normal distribution. Two sample t-test was applied for parametric data (ICP, MAP, CPP). Chi-square test was used for analysis of demographic data, localization, size and histopathologic diagnosis of the tumours, preoperative drug administration between the groups. Difference in dural tension and brain swelling were tested by chi-square test in 2×4 tables. For correlation, Pearson product moment correlation and linear regression were performed. Data were expressed as mean ±SD. *P*<0.05 was considered statistically significant.

## Results

A total of 68 patients were enrolled in the study with equal number of 34 patients in each group. Demographic data are shown in Table-1. There were no significant differences between the groups in terms of age, sex, weight and ASA grading.

**Table 1:** Demographic profile.

Variable	Group I	Group II
	Propofol	Isoflurane-N_2_O
**Age (Yr)**	52±12	51±14
**Sex(M/F)(n)**	18/16	20/14
**Body weight(kg)**	55±14	54±10
**ASA I/II**	28/6	26/8

Data are shown as number of patients or mean ±SD. *P*>0.05 No significant intergroup differences were observed.

Clinical data including time interval between induction and ICP measurement and vasopressor administration are shown in Table-2. There were no difference in neurological diagnosis (site, type and size of tumour) and number of patients taking antihypertensive or anticonvulsant. The number of patients requiring blood pressure support, differed significantly between groups and total dose of vasopressor used also was significantly higher in propofol group than isoflurane-nitrous oxide group.

**Table 2:** Clinical data

Variable	Group I	Group II
	Propofol	Isoflurane–N_2_O
**Lesion type**		
** Meningioma**	8	10
** Glioma**	12	15
** Metastasis**	8	5
** Others**	6	4
**Maximalvolume of tumour with oedema(cm^3^)**	105±108	120±138
**Midline shift (cm)**	1.1±1.8	1.2±2.4
**Preoperative antihypertensive (no. of patients)**	7	6
**Preoperative anticonvulsant (no. of patients)**	28	30
**Preoperative controlled diabetes mellitus (no.of patients)**	2	3
**MAP before induction(mmHg)**	103±12	105±14
**Phenylephrine administration (no. of patient)**	15	10*
**Time between induction and first ICP measurement (min)**	95±18	98±16

Data are shown as number of patients or mean ±SD.

* *P*<0.05 MAP=Mean arterial pressure.

After removal of bone flap subdural ICP, MAP were measured and CPP was derived and are summarized in Table-3. Before hyperventilation, there was no significant difference of MAP and CPP in between groups but subdural ICP was significantly lower in propofol group than isoflurane-nitrous oxide group. After hyperventilation ICP decreased significantly in isoflurane-nitrous oxide group. Difference in PaCO_2_ (before and after hyperventilation) was greater in propofol group but reduction in ICP after hyperventilation was significantly smaller as compared to isoflurane-nitrous-oxide group.

**Table 3:** Cerebral haemodynamics before and after hyper ventilation

	Group I	Group II
	Propofol	Isoflurane–N_2_O
**Before hyperventilation**		
** MAP(mmHg)**	75±12	88±14
** ICP(mmHg)**	8.5±4.6	14±6.9*
** CPP(mmHg)**	67±15	74±12
** PaCO2 (mmHg)**	35.5±3	34.5±3
**Afterhyperventilation**		
** MAP(mmHg)**	74±10	89±13
** ICP (mmHg)**	7.0±4.2	9.6±6.3
** CPP (mmHg)**	66±12	80±11*
** PaCO2 (mmHg)**	29.3±2.4	29.5±3

Data are shown as mean ±SD.

* *P*<0.05, Compared between groups.

^+^*P*<0.05, compared within group. MAP=Mean arterial pressure, ICP=Intracranial pressure, CPP=Cerebral perfusion pressure.

The dural tension before and after hyperventilation as estimated by neurosurgeon, are shown in Table 4. Before hyperventilation, dural tension was significantly higher in isoflurane-nitrous oxide group but after hyperventilation there was no significant difference in between groups. Degree of brain swelling after opening of dura, as shown in Table-5, was similar in both groups.

**Table 4:** Dural tension before and after hyper ventilation.

Variable	Propofol, n (%)	Isoflurane –N_2_O n(%)	P Value
**Dural tension before hyperventilation**			
VerySlack	4(11.8)	1(2.9)	
Normal tension	15(44.1)	10(29.4)	*P*<0.05
Increased Tension	13(38.2)	17(50)	
Pronounced tension	2(5.9)	6(17.6)	
**Dural tension after hyperventilation**			
VerySlack	6(17.6)	4(11.8)	
Normal tension	16(47)	14(41.8)	*P*>0.05
Increased Tension	10(29.4)	12(35.3)	
Pronounced tension	2(5.9)	4(11.8)	

Data are shown as number and percentage. Chi-square test (2×4 table) was applied for comparative analysis.

**Table 5:** Brain swelling score after opening of dura.

Brain Swelling score	Propofol n(%)	Isoflurane -N_2_O n(%)	*P* Value
No swelling	11(32.4)	8(23.5)	*P*>0.05
Minimal swelling	12(35.3)	13(38.2)	
Moderate swelling	10(29.4)	11(32.4)	
Pronounced swelling	1(2.9)	2(5.9)	

Data are shown as number and percentage. Chi-square test (2×4 table) was applied for comparative analysis.

There was a positive correlation between measured ICP and brain swelling score as well as neurological data (tumoursize and midline shift) and brain swelling score.

## Discussion

Different anaesthetic agents have different effects on cerebral haemodynamics. For example, propofol decreases CBF, ICP and may decrease cerebral perfusion pressure via its effects on blood pressure.^14^ Isoflurane appears to produce moderate increase in CBF and pronounced decrease in cerebral metabolism. An increase in ICP caused by it may be mild and can be prevented by hypocapnia^15^. Several groups have shown that it can increase ICP or decrease cerebral perfusion pressure in neurosurgical patients.^16^–^18^ Nitrous oxide is also a potent vasodilator and can increase CBF and ICP when given either alone or in combination with a volatile agent.^19^–^21^ But this increase can be attenuated by prior administration of thiopentone and hypocapnia^22^. Opioids are assumed to have no important effects as long as ventilation is controlled. Recently sufentanil, alfentanil and fentanyl have been reported to increase ICP and CBF^23^,^24^ although this has not been observed uniformly^25^ and all three drugs have been used successfully in neuroanaesthesia.

Despite these concerns, there is no clinical evidence that one particular anaesthetic management regimen is superior to other. The few available comparative trials suggest no important difference between propofol versus isoflurane, nitrous oxide versus no nitrous oxide.^2^,^26^,^27^ In the current study, we found that subdural ICP and the degree of dural tension were significantly lower during propofol anaesthesia as compared to isoflurane–nitrous oxide anaesthesia but after hyperventilation there was no statistical difference between two groups. Both before and after hyperventilation, a significantly lower CPP was found in propofol group. A significant difference was found regarding phenylephrine administration with regard to number of patients and total dose requirement.

In comparative studies of lumbar CSF pressure in patients without space occupying lesion subjected to desflurane, isoflurane, sevoflurane and propofol anaesthesia, a higher CSF pressure was found during volatile anaesthesia compared with propofol anaesthesia^8^,^16^. In another study of lumbar CSF pressure in neurosurgical patients subjected to either propofol-fentanyl or thiopental– isoflurane-fentanyl anaesthesia, no significant difference of lumbar CSF pressure was recorded^2^.

Subdural pressure is more accurate as a regional estimate^10^,^11^ compared with lumbar CSF pressure measurement because tumour or cerebral edema localized close to craniotomy increases subdural pressure more than lumbar CSF pressure. In addition, obliteration of CSF pathway caused by tumour makes lumbar CSF pressure less reliable. No significant difference in epidural ICP was recorded in another comparative study of propofol and isoflurane–nitrous oxide anaesthetized patients.^1^ In principle, their findings corroborate with those of current study. Methodologic differences in ICP monitoring might explain the comparatively lower ICP values in our study.

In this study, brain swelling after opening of dura was similar in both groups. Neurosurgeons were blinded to anaesthetic technique. In another study no difference was mentioned regarding brain swelling between isoflurane-N_2_O and propofol/fentanyl group^1^. However, a significant correlation was found between brain swelling score and ICP.

In clinical studies, cerebral autoregulation is preserved with propofol but is impaired during 1.5 MAC isoflurane.^28^,^29^ As such, a higher CPP during isoflurane-nitrous oxide anaesthesia should elicit a decrease in ICP. The question of whether cerebral autoregulation influences ICP is further complicated by the fact that cerebral autoregulation is impaired or abolished in patients with cerebral tumours.^30^ In this study, isoflurane administration was restricted to well defined and low MAC level (1 MAC) and maintenance doses of propofol and fentanyl were well defined. Accepted mean arterial blood pressure reduction was 20%, otherwise vasopressor was administered.

In this study, the decrease in ICP after hyperven-tilation averaged 1.5mmHg in the propofol group but 3mmHg in isoflurane-N_2_O group. The significantly greater decrease in ICP during isoflurane-nitrous oxide anaesthesia may be due to better carbon dioxide responsiveness. This is in agreement with other clinical studies indicating a preserved carbon dioxide reactivity during anaesthesia with isoflurane^4^,^31^ but decreased carbon dioxide reactivity during anaesthesia with propofol.^32^,^33^

It is tempting to conclude that one anaesthetic regimen is better than other. Each anaesthetic has advantages and disadvantages. As noted, isoflurane and nitrous oxide are vasodilators and both can increase ICP. In contrast, total intravenous anaesthesia consisting of propofol and fentanyl (without nitrous oxide) should reduce CBF and ICP. But combination of low dose isoflurane with nitrous oxide and fentanyl might cause an intermediate increase in CBF and ICP because concentration of either drug can be decreased than concentration of any agent when using alone to provide an anaesthetic plane required for surgical resection. None of the anaesthetic was associated with any intra operative difficulties.

We designed the current trial in such a manner that the subject population was as uniform as possible and we restricted the trial to patients with known supratentorial tumours with midline shift less than 10mm. Although supratentorial surgery in patients with large tumour might reveal differences, in this study, any significant difference in subdural ICP and brain condition for surgery after hyperventilation was not revealed in both groups. Limitation of the study were firstly inability to estimate carbon dioxide reactivity, inability to define absolute end points of anaesthesia in both groups in absence of anaesthetic depth monitor.

The current study indicates that during craniotomy for supratentorial cerebral tumours, subdural ICP is lower in patients anaesthetized with propofol than isoflurane-nitrous oxide. After hyper ventilation, there were no significant difference of ICP and dural tension. Degree of brain swelling after opening of dura was similar in both groups. CPP was higher, may be due to better preservation of carbon dioxide responsiveness in isoflurane-nitrous oxide group as compared with propofol group. These results support the view that despite their cerebrovascular effects, institution of hyperventilation makes both anaesthetic regimens equally acceptable for intracranial tumour surgery.
